# Physiological aspects of the determination of comprehensive arterial inflows in the lower abdomen assessed by Doppler ultrasound

**DOI:** 10.1186/1476-7120-10-13

**Published:** 2012-03-26

**Authors:** Takuya Osada

**Affiliations:** 1 Department of Sports Medicine for Health Promotion, Tokyo Medical University, Tokyo, Japan; 2 Cardiac Rehabilitation Centre, Tokyo Medical University Hospital, Tokyo, Japan

**Keywords:** Lower abdominal inflows, splanchnic blood flow, Doppler ultrasound

## Abstract

Non-invasive measurement of splanchnic hemodynamics has been utilized in the clinical setting for diagnosis of gastro-intestinal disease, and for determining reserve blood flow (BF) distribution. However, previous studies that measured BF in a "single vessel with small size volume", such as the superior mesenteric and coeliac arteries, were concerned solely with the target organ in the gastrointestinal area, and therefore evaluation of alterations in these single arterial BFs under various states was sometimes limited to "small blood volumes", even though there was a relatively large change in flow. BF in the lower abdomen (BF_Ab_) is potentially a useful indicator of the influence of comprehensive BF redistribution in cardiovascular and hepato-gastrointestinal disease, in the postprandial period, and in relation to physical exercise. BF_Ab _can be determined theoretically using Doppler ultrasound by subtracting BF in the bilateral proximal femoral arteries (FAs) from BF in the upper abdominal aorta (Ao) above the coeliac trunk. Prior to acceptance of this method of determining a true BF_Ab _value, it is necessary to obtain validated normal physiological data that represent the hemodynamic relationship between the three arteries. In determining BF_Ab_, relative reliability was acceptably high (range in intra-class correlation coefficient: 0.85-0.97) for three arterial hemodynamic parameters (blood velocity, vessel diameter, and BF) in three repeated measurements obtained over three different days. Bland-Altman analysis of the three repeated measurements revealed that day-to-day physiological variation (potentially including measurement error) was within the acceptable minimum range (95% of confidence interval), calculated as the difference in hemodynamics between two measurements. Mean BF (ml/min) was 2951 ± 767 in Ao, 316 ± 97 in left FA, 313 ± 83 in right FA, and 2323 ± 703 in BF_Ab_, which is in agreement with a previous study that measured the sum of BF in the major part of the coeliac, mesenteric, and renal arteries. This review presents the methodological concept that underlies BF_Ab_, and aspects of its day-to-day relative reliability in terms of the hemodynamics of the three target arteries, relationship with body surface area, respiratory effects, and potential clinical usefulness and application, in relation to data previously reported in original dedicated research.

## Background

The splanchnic circulation has been described as the "blood-giver of circulation" and is believed to play a major role in overall cardiovascular regulation [1]. The splanchnic system receives nearly 30% of the cardiac output through three large arteries: the coeliac and the superior and inferior mesenteric arteries. Hemodynamics in the splanchnic organs are altered under various stressful conditions, such as during physical activity and in the postprandial state [2], due to balancing of tone between sympathetic and vagus activity; consequently, clinical assessment of the splanchnic circulation could potentially provide valuable information regarding hepato-gastrointestinal disease [3-5] and cardiac dysfunction [6].

Previous Doppler ultrasound studies that measured splanchnic blood flow in a "single vessel with small size volume", such as the superior mesenteric, coeliac artery, or portal vein, were concerned solely with the target organ in the gastrointestinal area [2,7-9]; therefore, evaluation of alterations in these single arterial blood flows under the various states were sometimes limited to small volumes, even though there was a relatively large change in flow. Evaluation of the comprehensive arterial blood flow in the lower abdomen (BF_Ab_), including the liver, spleen, gastro-intestine, kidney, and pelvic organs as a multiple arterial function, may potentially be a feasible method of determining the distribution of abdominal blood-flow volume or disorder in cases of splanchnic or cardiovascular dysfunction, as well as the distribution following nutritious meal intake or physical exercise [10,11].

Our previous studies used ultrasonography to assess whole arterial BF_Ab _hemodynamics: BF_Ab _was obtained by subtracting blood flow in the bilateral proximal femoral arteries [left femoral artery (LFA) and right femoral artery (RFA)] from blood flow in the upper abdominal aorta (Ao) above the coeliac artery bifurcation [10-14].

This method of quantitative assessment is a challenging but unique and non-invasive procedure for determining the comprehensive inflows of all abdominal organs, and is a potentially useful indicator of blood flow redistribution in cardiovascular and hepato-gastrointestinal disease, in the postprandial period, and in relation to physical exercise.

Variability in the hemodynamics (blood velocity, vessel diameter and blood flow) of the three target arteries is valuable information for determining BF_Ab_. Therefore, the purpose of the present review is to summarize the methodology for determining BF_Ab _using validated data of three target arteries, to discuss methodological considerations and limitations in view of previously reported findings, and to consider the potential clinical usefulness and application of measurements for the comprehensive lower abdominal flows.

## Methodology

### Subjects

The subjects were the participants of three studies: 60 healthy males (mean age, 24.1 ± 5.5 years; mean height, 173.3 ± 7.1 cm; and mean body weight, 68.5 ± 9.3 kg), 50 healthy males (mean age, 23.5 ± 4.9 years; mean height, 172.8 ± 7.1 cm; and mean body weight, 67.6 ± 9.9 kg) and 10 healthy males (mean age, 25.2 ± 6.6 years; mean height, 175.6 ± 7.0 cm; and mean body weight, 70.1 ± 7.6 kg). All values are expressed as mean ± standard deviation (SD). Participants had no previous history of cardiovascular disease, gastrointestinal disease, hypertension, or anaemia, and no abnormality of the peripheral vasculature. The studies were conducted according to the principles of the Declaration of Helsinki (1976) and with the approval of the Institutional Ethics Committee of the author's institution. All participants gave their written consent and were informed of the nature and purpose of the study, as well as potential risks and discomfort. The participants also understood that they could withdraw from the study at any time without consequence. The study populations reviewed in the present study do not include the elderly.

### Approach for Doppler ultrasound assessment of three target arteries for determining BF_Ab_

**Figure 1:**
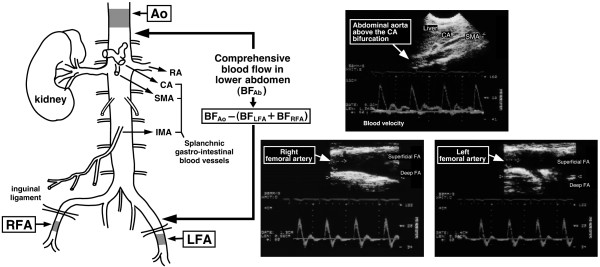
**Anatomical region for measurement and blood velocity profiles of the three arteries**. Blood flow (BF) measurements were obtained for the upper abdominal aorta (Ao) above the coeliac artery bifurcation and for the bilateral femoral arteries (right and left femoral arteries; RFA and LFA, respectively). Comprehensive BF in the lower abdomen (BF_Ab_) was calculated by subtracting bilateral femoral arterial flow (BF_LFA _+ BF_RFA_) from BF_Ao_. The splanchnic gastrointestinal blood vessels include the renal (RA) and coeliac arteries (CA), and the superior and inferior mesenteric arteries (SMA and IMA, respectively). Figure modified from Osada *et al. *[12], reproduced with permission from IOP Publishing Ltd.

The target vessels were the following three conduit arteries: 1) the Ao at ~3 cm above the coeliac artery bifurcation, 2) the proximal LFA, and 3) the proximal RFA (Figure 1). The Ao region was most commonly measured just below the diaphragm in longitudinal section view (from the sub-sternal area) to enable Ao sample volume to be maintained at the end of the expiratory phase during spontaneous breathing. Detection of the Ao was relatively constant and free from interference from intestinal gas. For both femoral arteries, measurement location was chosen to minimize turbulence and the influence of the inguinal region on blood flow above the bifurcation, thereby enabling easy and reliable measurement [10-18]. Blood velocity (pulsed wave) and vessel diameter (2-dimensional) measurements were obtained using a curvilinear array probe (3.5 MHz) for Ao and a linear array probe (7.5 MHz) for the LFA and RFA. The insonation angle was maintained below 60° for each participant and remained constant throughout the experiments [10-14,19]. The sample volume was placed in the precise centre of the vessel before being adjusted to cover the width of the vessel diameter and blood velocity distribution.

The data in the present review were obtained using an ultrasound unit (SONOS 1500, HP77035A; Hewlett-Packard, Tokyo, Japan) with a real-time two-dimensional ultrasonic imager and a pulsed-Doppler flowmeter for calculating maximum envelope in the blood velocity profile. The Doppler instrument used in this review, however, could not determine time- and spatial-averaged and amplitude (signal intensity)-weighted mean blood velocities; thus, the measured blood velocity determined by integration of the outer envelope (maximum velocities) would have reflected the higher (maximum) velocity component at the centre of the vessel through the cardiac cycle. Because this procedure takes no account of the lower velocity component of the flow profile, the blood velocity values in this review have potentially been overestimated. On the basis of this physiological phenomenon, the above-mentioned measure of mean blood velocity, which expresses the averaged speed for all red blood cells within the vessel, is more precise; however, the present procedure used to determine blood velocity also provides acceptable data.

Recent developments in ultrasound instrumentation include an auto-tracing programme for determining mean and maximum blood velocity (outer envelope). Maximum (envelope) blood velocity in the femoral artery is previously reported as being ~1.53 [20] and 1.3-1.8 [21,22] times higher than mean blood velocity. An in vitro study that used a silicon tube to model the conduit artery found that maximum (envelope) blood velocity was approximately 1.75 times higher than mean blood velocity [23].

### Measurement procedure of blood velocity, vessel diameter, and blood flow in the arteries

For each participant, measurement was conducted between approximately 7 and 9 am, while fasting. Prior to measurement, colour Doppler was used to check for unsuspected pathology in each conduit artery. For each of the three conduit arteries, blood velocity was measured for 2-3 minutes and vessel diameter for 1-2 minutes via image observation using a wide expanded view in longitudinal section, by a single operator (the author). Blood velocity was analysed by integrating the outer envelope of the maximum velocity values from the flow profile for each beat, for approximately 20-40 beats [10,24-26]. Blood velocity and vessel diameter analyses were performed using the phase that demonstrated similar heart rate and blood pressure values among measurements from the three conduit arteries. The systolic and diastolic vessel diameter of each conduit artery was measured in relation to the electrocardiograph displayed on the monitor of the ultrasound unit.

Vessel diameter was also measured under perpendicular insonation and calculated in relation to the temporal duration of the electrocardiography recording curve, as follows: [(systolic vessel diameter × 1/3) + (diastolic vessel diameter × 2/3)] [15-17]. The mean vessel diameter for each beat was calculated over approximately 20-40 beats. Blood flow was determined by multiplying the cross-sectional area [area = π × (vessel diameter/2)^2^] by the amplitude (signal intensity)-weighted blood velocity (time- and spatial-averaged outer envelope of the maximum velocity). To determine BF_Ab _precisely, blood pressure and heart rate should remain in a steady state during measurement of the three target arteries.

### Determination of comprehensive BF_Ab_

Blood flow in the Ao, LFA, and RFA is defined as BF_Ao_, BF_LFA_, and BF_RFA_, respectively. BF_Ab _was calculated by subtracting the sum of BF_RFA _and BF_LFA _from BF_Ao_, as follows: BF_Ab _= [BF_Ao _- (BF_LFA _+ BF_RFA_)] [10-14].

## Consideration of the physiological aspect of the three arterial blood flows and BF_Ab_

### Day-to-day reliability and variability in hemodynamics of the three arteries and BF_Ab _via repeated measurements

Hemodynamic measurements (blood velocity, vessel diameter, and blood flow) in the three arteries were performed on three consecutive days by a single operator. BF_Ab _can then be determined for each day by the formula BF_Ao _- (BF_LFA _+ BF_RFA_), with blood flow calculated by multiplying blood velocity by the cross-sectional area. Variability in the values of blood velocity, vessel diameter, and blood flow in the three arteries may be valuable information for determining BF_Ab_.

First, relative reliability was estimated by analysing the three hemodynamic measurements repeated on three different days, for 60 healthy male participants [12]. As shown in Table 1, F-test revealed no significant difference in blood velocity, vessel diameter, or blood flow values among the three measurements. Consequently, the single-measure intra-class correlation coefficient was significantly high for relative reliability estimated by repeated hemodynamics measurements [27]. This indicates that Doppler assessment of hemodynamic parameters in the three target arteries has potential as a stable and acceptable procedure for determining BF_Ab_.

**Table 1:** Reliability and coefficients of variation in hemodynamics for repeated measurements

Hemodynamics variable	Target artery	1st		2nd		3rd		Mean CV (%)	Relative reliability, SM-ICC	*F *(2, 118, 0.05)	*p *< 0.05
						
		Mean	SD	Mean	SD	Mean	SD				
Blood velocity (cm/sec)	Ao	26.0	6.6	26.2	6.3	26.0	7.0	4.9	0.94	0.18	ns
	LFA	8.4	2.5	8.3	2.3	8.2	2.5	7.8	0.90	0.48	ns
	RFA	8.4	2.5	8.2	2.2	8.3	2.6	8.7	0.85	1.15	ns

Vessel diameter (mm)	Ao	15.5	1.3	15.6	1.2	15.5	1.2	1.3	0.95	3.63	ns
	LFA	9.0	0.7	9.1	0.7	9.0	0.7	1.4	0.94	3.09	ns
	RFA	9.1	0.8	9.1	0.7	9.0	0.8	1.2	0.97	1.35	ns

Blood flow (ml/min)	Ao	2946	774	2989	740	2931	818	4.9	0.95	1.65	ns
	LFA	322	104	324	105	317	113	8.6	0.90	0.66	ns
	RFA	323	103	317	94	319	106	8.6	0.90	0.57	ns
	Ab	2301	699	2348	666	2295	721	6.2	0.94	1.87	ns

Mean coefficients of variation (CV) in hemodynamics were obtained from the average CV values of 60 participants among three repeated measurements over three different days. Single-measure intra-class correlation coefficient (SM-ICC) was evaluated by three repeated measurements over three different days. All measurements were performed by a single operator. SD, standard deviation; ns, not significant; Ao, abdominal aorta; LFA, left femoral artery; RFA, right femoral artery; Ab, lower abdomen. This material is reproduced and additional data were provided for analysis from Osada *et al. *[12], with permission from IOP Publishing Ltd.

**Figure 2:**
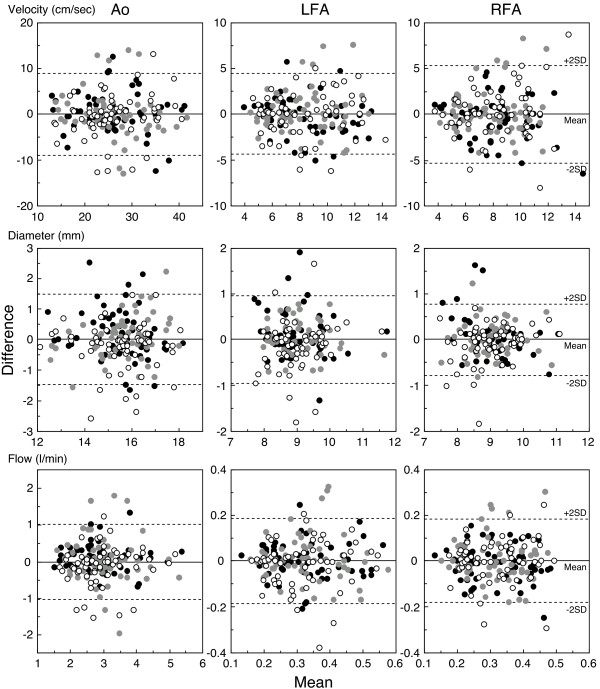
**Bland-Altman analysis of three arterial hemodynamics for repeated measurement**. The difference (y-axis) and mean (x-axis) are shown for hemodynamics (blood velocity, vessel diameter, and blood flow, respectively) in the three arteries between two measurements obtained over three different days. Results are based on 180 samplings (comparison between 1^st ^and 2^nd^, 2^nd ^and 3^rd^, and 3^rd ^and 1^st ^measurements) in 60 participants. The solid line indicates bias (close to zero) and the dashed lines are the limits of agreement of ± 1.96 SD (± 2 SD on the figure). The open circles correspond to 1^st ^*vs*. 2^nd ^measurement. The grey circles correspond to 2^nd ^*vs*. 3^rd ^measurement. The closed circles correspond to 3^rd ^*vs*. 1^st ^measurement. All measurements were obtained by a single operator, using the same ultrasound instrument. Thus, no systematic bias (fixed bias or proportional bias) exists between two measurements. Ao, abdominal aorta; LFA, left femoral artery; RFA, right femoral artery. Material reproduced with additional data in the adapted figure, with Bland-Altman analysis, from Osada *et al. *[12], with permission from IOP Publishing Ltd.

**Figure 3:**
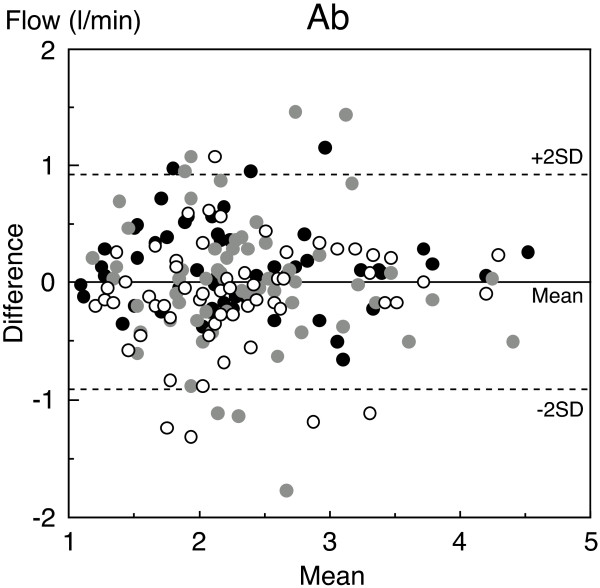
**Bland-Altman analysis of blood flow in the lower abdomen for repeated measurements**. Bland-Altman analysis revealed no systematic bias (fixed bias or proportional bias) between two measurements. The difference (y-axis) and mean (x-axis) in blood flow in lower abdomen (Ab) between two measurements over three different days. Results are based on 180 samplings (comparison between 1^st ^and 2^nd^, 2^nd ^and 3^rd^, and 3^rd ^and 1^st ^measurements) in 60 participants. The solid line indicates bias and the dashed lines are the limits of agreement of ± 1.96 SD (± 2 SD on the figure). The open circles correspond to 1^st ^*vs*. 2^nd ^measurement. The grey circles correspond to 2^nd ^*vs*. 3^rd ^measurement. The closed circles correspond to 3^rd ^*vs*. 1^st ^measurement. Material reproduced, with data added to the figure adapted from Osada *et al. *[12], with permission from IOP Publishing Ltd.

Second, Bland-Altman analysis [28] was used for statistical analysis of mean values (x-axis) and difference values (y-axis) in hemodynamics (blood velocity, vessel diameter, and blood flow) between two measurements over three different days (Figure 2). Figure 2 shows that no systematic bias (fixed bias and proportional bias) was found between any two measurements over three different days. The limits of agreement (mean ± 1.96 SD) in terms of the difference between two hemodynamics measurements and the 95% confidence interval also indicate validity in the present study population within an acceptable and permissible range and true mean values (Table 2). Bland-Altman analysis revealed that the acceptable range in difference (bias) between two measurements may be less than 8.9 cm/s for Ao and less than 5.3 cm/s for blood velocity in the femoral arteries; less than 1.5 mm for Ao and less than 0.95 mm for vessel diameter in the femoral arteries; and less than 1.0 l/min and less than 0.18 l/min for blood flow in the Ao and femoral arteries, respectively. It is considered that this range takes into account day-to-day physiological variation as well as measurement error. The present results for repeated measurements on three different days reveals that the range in blood flow values in the three arteries remained similar for individual participants under similar testing conditions; thus, mean BF_Ab _is considered a reliable value with the acceptable range in difference (bias) between two measurements may be less than 0.9 l/min for blood flow in the Ab. (Tables 1, 2; Figure 3).

**Table 2:** Bland-Altman analysis of acceptable range in difference (bias) in hemodynamics via three repeated measurements

Hemodynamics variable		Ao	LFA	RFA	Ab
Blood velocity (cm/sec)	Limit of agreement	0.00 ± 8.93	0.00 ± 4.37	0.00 ± 5.24	
	95%CI	0.00 ± 0.67	0.00 ± 0.33	0.00 ± 0.39	

Vessel diameter (mm)	Limit of agreement	0.00 ± 1.49	0.00 ± 0.95	0.00 ± 0.77	
	95%CI	0.00 ± 0.11	0.00 ± 0.07	0.00 ± 0.06	

Blood flow (ml/min)	Limit of agreement	0.00 ± 1008.42	0.00 ± 186.78	0.00 ± 180.71	0.00 ± 922.08
	95%CI	0.00 ± 75.16	0.00 ± 13.92	0.00 ± 13.47	0.00 ± 68.73

Results are based on 180 samplings (comparison between 1^st ^and 2^nd^, 2^nd ^and 3^rd^, and 3^rd ^and 1^st ^measurements) in 60 participants. All measurements were performed by a single operator. The mean difference (bias) in each hemodynamic parameter (blood velocity, vessel diameter, and blood flow) between two measurements almost corresponds to zero. Therefore, the limit of agreement is expressed as 0.00 ± 1.96 SD. The 95% of confidence interval (95%CI) is expressed as 0.00 ± 1.96 SE. Ao, abdominal aorta; LFA, left femoral artery; RFA, right femoral artery; Ab, lower abdomen; SD, standard deviation; SE, standard error. This material is reproduced and additional data were provided for analysis from Osada *et al. *[12], with permission from IOP Publishing Ltd.

### Validity of target arterial blood flows and BF_Ab _compared with previous findings

#### Abdominal aorta

Values for vessel diameter, estimated cross-sectional area, and blood flow of the upper abdominal aorta obtained in the present review were 15.6 ± 1.2 mm, 1.91 ± 0.29 cm^2^, and 2951 ± 767 ml/min, respectively (Table 3).

**Table 3:** Mean values and range for the three arterial hemodynamics and lower abdominal blood flow

Hemodynamics variable		Ao	LFA	RFA	Ab
Blood velocity (cm/sec)	Mean ± SD	26.1 ± 6.5	8.3 ± 2.4	8.1 ± 2.1	--
	Range	13.9–41.1	4.9–13.4	4.0–12.7	--
	95%CI	24.4–27.7	7.7–8.9	7.6–8.7	--

Vessel diameter (mm)	Mean ± SD	15.6 ± 1.2	9.0 ± 0.7	9.1 ± 0.7	--
	Range	12.4–18.1	7.9–11.6	7.6–10.9	--
	95%CI	15.2–15.9	8.9–9.2	8.9–9.2	--

Blood flow (ml/min)	Mean ± SD	2951 ± 767	316 ± 97	313 ± 83	2323 ± 703
	Range	1585–5274	178–558	168–481	1153–4401
	95%CI	2757–3145	291–340	292–334	2145–2500

Mean values are shown for three repeated measurements over three different days in 60 participants. All measurements were performed by a single operator. Ao, abdominal aorta; LFA, left femoral artery; RFA, right femoral artery; Ab, lower abdomen; 95%CI, 95% of confidence interval; SD, standard deviation. This material is reproduced and additional data were provided for analysis from Osada *et al. *[12], with permission from IOP Publishing Ltd.

The present values for diameter and cross-sectional area are similar to those of 15.5-17.6 mm and 1.88-2.43 cm^2^, respectively, measured by Gabriel and Kindermann [29]. Nimura et al. [7] reported blood flow values obtained by Doppler ultrasound of the upper abdominal aorta and the sum total blood flow of the coeliac, superior mesenteric and both renal arteries as 2470-3246 ml/min and 2450-3549 ml/min, respectively. These values are similar to those for BF_Ao _expressed in the present review.

#### Femoral artery

The present values for the diameter of the femoral arteries were 9.0 ± 0.7 mm in the LFA and 9.1 ± 0.7 mm in the RFA (Table 3), which is in the same range as the previously reported values of 7.5 ± 0.3 mm measured using angiography [30] and 8.1 ± 0.11 mm measured by Duplex Doppler [31]. In the present review, cross-sectional area of the femoral arteries was 0.65 ± 0.1 cm^2 ^in the LFA and 0.65 ± 0.11 cm^2 ^in the RFA. Mean blood velocity in the femoral arteries was 8.3 ± 2.4 cm/s in the LFA and 8.1 ± 2.1 cm/s in the RFA; these values are in the same range as that of 10.2 ± 0.39 cm/s previously measured by pulsed Doppler [31]. Blood flow in the femoral arteries was 316 ± 97 ml/min in the LFA and 313 ± 83 ml/min in the RFA. These values are in the same range as those of 450-886 ml/min [32], 301 ± 81 ml/min [33], and 390 ± 20 ml/min [34] measured using indicator dilution; and 376 ± 154 ml/min [35], 226.5 ± 28.6 ml/min [31], 344 ml/min [36], and 350-367 ml/min [37] measured by Doppler ultrasound. Furthermore, Ganz et al. [38] reported a value of 383-766 ml/min by thermodilution, and Vänttinen [39] reported a value of 239 ml/min using electromagnetic flowmetry. These values are in the same range in those of the present review, despite differences in the method of measurement. Blood flow may also be influenced by the subject's position during measurement and local blood flow per body weight, as well as thigh muscle mass [40].

#### Blood flow in the lower abdomen

There is lack of comparative BF_Ab _data measured by other valid methods (gold standard) such as the thermodilution technique or the cardiovascular magnetic resonance method. However, ultrasound Doppler is also an acceptable valid measure for determining blood velocity/flow in the conduit artery.

Including the results of previous reports [12], the range of BF_Ab _over the three different days was 1153-4401 ml/min in the 60 participants. Furthermore, the mean value of BF_Ab _was 2630 ± 649 ml/min in 18 of the participants (age range, 20-38 years) in the previous reports [10].

Based on the general anatomical features shown in Figure 1, the BF_Ab _values are considered to indicate the sum of blood flow to the coeliac artery; mesenteric arteries; the bilateral renal, suprarenal, gonadal, and internal iliac arteries; and some lumbar arteries.

Previous studies [41-44] reported average splanchnic blood flow (including that of the coeliac trunk, superior mesenteric, and inferior mesenteric arteries) as approximately 1500 ml/min, corresponding to 20%-30% of cardiac output. The sum of the blood flow values in the two renal arteries is approximately 1000-1200 ml/min, which corresponds to 20% of cardiac output [45]. In addition, blood flow is 1400 ml/min in the liver, gastro-intestine, and spleen (the so-called splanchnic organs), and 1100 ml/min in the kidney [46]. The range of values for the sum of blood flow in the "splanchnic" and the "two renal arteries" reported in previous studies is similar to the BF_Ab _values obtained using the present method. However, the wide range in BF_Ab _may also be related to individual physical features such as body surface area and body weight.

### Relationship of blood flows to body surface area and to body weight

Previous studies have shown that cardiac output increases in proportion to body surface area [46,47], which means that cardiac output is regulated throughout life in almost direct proportion to overall metabolic activity. Furthermore, a positive correlation has been demonstrated between cardiac output and abdominal-splanchnic blood volume, using whole-body scintigraphy [48]. BF_Ab _is expected to be closely related to body surface area, because the splanchnic system receives ~30% of cardiac output.

**Figure 4:**
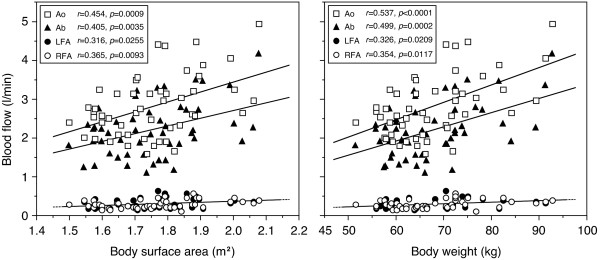
**Relationship of blood flows to body surface area and to body weight**. A significant (*p *< 0.05) positive linear correlation was observed between measured blood flow, and body surface area and body weight for Ao, LFA, RFA, and Ab (*n *= 50). Note overlapping of the circular regression lines for blood flow in LFA and RFA. Ao, abdominal aorta; LFA, left femoral artery; RFA, right femoral artery; Ab, lower abdomen. Figure adapted from Osada *et al. *[13], reproduced with permission from The International Science Literature, Inc.

A significant positive relationship exists between BF_Ab _and both body surface area and body weight (Figure 4). The formula used to calculate body surface area is widely used in the target population [49]. Furthermore, an increase in BF_Ab _with increasing body weight may be reasonable, taking into consideration the total weight of the lower abdomen. This relationship is based on the concept that blood flow distribution is associated with a higher flow per weight to the liver and intestine compared with skeletal muscle at rest [50]. An expected additional finding was that peripheral blood flow at each conduit artery also had a positive linear relationship (*p *< 0.05) with body surface area as well as with body weight (Figure 4). This correlation is in agreement with evidence concerning the relationship between cardiac output supply and peripheral arterial blood flow, with cardiac output being closely related to body surface area [46,47].

### Relationship of BF_Ab _to BF_Ao_, BF_LFA_, and BF_RFA _in estimating BF_Ab_

The distribution of BF_Ab _may be influenced by the magnitude of both cardiac output and limb blood flows. Specifically, it is speculated that alterations in limb blood flow may play an important role in regulating BF_Ab _via changes in the tone in the vascular bed of abdominal organs during low-intensity exercise when there is little fluctuation in the magnitude of BF_Ao _[10]. Similarly, it is unclear whether BF_Ab _or cardiac output at rest has a major impact on limb blood flow in a steady state of neural response.

**Figure 5:**
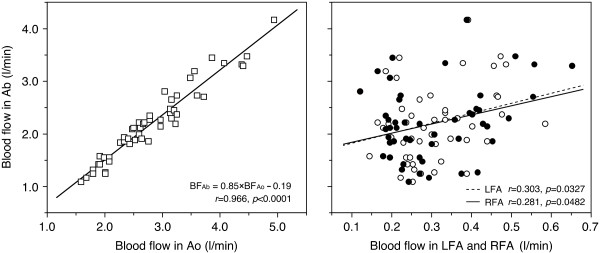
**Relationship of blood flow in lower abdomen to the three arteries**. BF_Ab _was more strongly related to BF_Ao _(*r *= 0.966, *p *< 0.0001) than to BF_LFA _or BF_RFA _(*r *= 0.303, *p *= 0.0327 in LFA; *r *= 0.281, *p *= 0.0482 in RFA) (*n *= 50). Ao, abdominal aorta; LFA, left femoral artery; RFA, right femoral artery; Ab, lower abdomen. Blood flow in the Ao, LFA, RFA and Ab is defined as BF_Ao_, BF_LFA_, BF_RFA_, and BF_Ab _respectively. Figure adapted from Osada *et al. *[13], reproduced with permission from The International Science Literature, Inc.

Day-to-day coefficients of variation in blood flow were relatively high in the femoral arteries compared with BF_Ao_, even though the absolute BF_Ao _values were approximately 10 times higher than those of blood flow in both femoral arteries. Accordingly, BF_Ab _was more strongly related to BF_Ao _(*r *= 0.966) than to BF_LFA _(*r *= 0.303) or BF_RFA _(*r *= 0.281) (Figure 5). Alterations in BF_Ao _that are closely related to cardiac output (except cerebral and arm blood flows) potentially have the greatest influence on BF_Ab_, even if blood flow in the femoral arteries has less influence on BF_Ab_, at least at rest; accordingly, BF_Ao _potentially has the largest influence on BF_Ab _as a central hemodynamic factor. Figure 5 shows that precise BF_Ab _values may be unreliable when there are large variations in both BF_LFA _and BF_RFA_. Thus, evaluation of BF_Ab _may be better expressed by the following formula: BF_Ab _(l/min) = 0.85 × BF_Ao _- 0.19, if Ao measurement alone is performed (Figure 5).

### Effect of respiration and posture

Deep thoracic breathing in inspiration produces rapid acceleration of blood flow in veins located near the thorax, such as the hepatic vein, jugular vein, and inferior vena cava [51], while the blood velocity in these veins is reduced just as rapidly at the start of expiration [52]. Mechanical ventilation causing a higher positive end-expiratory pressure-induced increase in lung volume could impede venous return, thereby altering systemic hemodynamics and hepatic venous outflow [53]. In animal experiments, portal vein blood velocity and hepatic arterial blood velocity were shown to decrease with positive end-expiratory pressure as a result of a simple increase in the downstream pressure [54].

**Figure 6:**
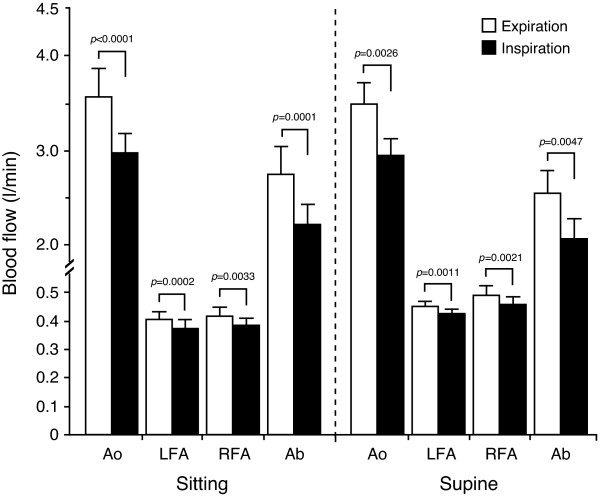
**Blood flow in the three arteries and lower abdomen in relation to respiration and posture**. Blood flow was significantly less in inspiration compared with expiration in Ao, LFA, and RFA, in both sitting and supine positions. Consequently, blood flow in Ab was lower in inspiration than in expiration, in both sitting and supine positions (*n *= 10). Values are expressed as the mean ± standard error (SE). Ao, abdominal aorta; LFA, left femoral artery; RFA, right femoral artery; Ab, lower abdomen. Figure adapted from Osada *et al. *[14], reproduced with permission from The International Science Literature, Inc.

Also relevant to the present method are respiratory phase and posture, which are related to alteration in BF_Ab_, as shown in Figure 6. The difference in BF_Ab _was approximately 550 ml/min between inspiration and expiration in the sitting position; in the supine position, the difference was 480 ml/min. Blood flow was significantly less in inspiration compared with expiration in Ao, LFA, and RFA, in both the sitting and supine positions. BF_Ab _was found to be lower in inspiration than in expiration, in both the sitting and supine positions. Respiration-related changes in the hemodynamics of the three conduit arteries potentially lead to alterations in BF_Ab_. This result may be in partial agreement with the theory that respiratory-induced alteration of BF_Ab _occurs with impedance of venous return in the splanchnic area.

The change in intra-abdominal pressure during breathing (thoracic-abdominal movement) possibly reflects transient changes in blood velocity in the Ao and femoral arteries. Higher values of venous outflow are found in the hepatic and portal veins in the supine rather than upright position, due to the effect of gravity [51]. In contrast, the reduction of BF_Ab _in the inspiratory phase is similar between sitting and supine, and thus no postural effect on BF_Ab _is seen in Figure 6. As the splanchnic circulation is intrinsically susceptible to the adverse effects of hydrostatic force [51,55], redistribution due to postural change between sitting and supine may differ between venous and arterial sites. Respiratory effects should be taken into account in evaluation of BF_Ab _determined by measurements obtained in the three arteries. The present study demonstrated that changes in blood velocity between expiration and inspiration in the three conduit arteries may potentially indicate alterations in BF_Ab_, and are only minimally influenced by posture. This phenomenon could be due to mechanical compression of vascular flow perfusion in comprehensive BF_Ab _or via the vasovagal response. Because respiration and posture effects have an effect when organ perfusion is adequate, it is important not to confuse these effects as a sign of impaired organ perfusion. Evaluation of BF_Ab _hemodynamics in the three conduit arteries should take respiratory effects into account.

### Limitations

The disadvantages of the present methods are that measurement of the three target arteries cannot easily be performed in a short period of time, and that blood flow in the pelvis and other organs (except for the target splanchnic area) cannot be excluded. To avoid over- or underestimation of BF_Ab_, measurement of the three target arteries should be performed under steady-state conditions at rest, with only minor changes in heart rate and blood pressure.

### Potential clinical usefulness and application

Evaluation of BF_Ab _as a quantitative assessment, encompassing physiologic flow, is a potentially useful indicator of 1) reserve blood volume and 2) blood flow in redistribution in the lower abdominal circulation in cardiovascular and hepato-gastrointestinal disease, shock, multiple organ failure, and stressful conditions such as following physical exercise and in the postprandial period. The advantage of the present method is that it enables evaluation of comprehensive BF_Ab _without interference from intestinal bowel gas, because the three target conduit arteries can be detected relatively easily. It may also be useful for examining pathological hemodynamics, which may influence the abdominal circulation under the conditions of 1) extraordinary hemodynamics associated with abdominal aneurysm; 2) collateral circulation in abdominal-iliac peripheral arterial disease, as well as comparison with the post-operative state; and 3) intestinal neurological dysfunction associated with spinal disorder, in cerebrovascular disease, and in orthostatic hypotension with vasovagal syncope.

Although Doppler methods are less commonly used for quantifying flow in the three target arteries compared with other techniques, the measurement procedure used in the present method is potentially clinically viable. However, because it can be time-consuming to perform routine hemodynamic measurements for the three conduit arteries, measurement may be limited to the region of the femoral arteries around the inguinal ligament close to the genital area, which may not be an acceptable method for general use. It is possible that Doppler ultrasound evaluation of BF_Ab _using a single vessel (Ao) may enable the necessary information to be obtained (Figure 5).

Also of note, because BF_Ab _values are potentially related to many factors, including mean arterial blood pressure and/or cardiac index, it has potential use as a surrogate parameter for central venous saturation in the clinical setting.

## Conclusions

The advantage in the described procedure for the determination of splanchnic hemodynamics is that it may potentially enable evaluation of the whole lower abdominal blood flows, assessed by non-invasive measurement using cardiovascular ultrasound. In contrast, it has the disadvantage that measurement of the three target arteries during steady heart rate and blood pressure can be time-consuming, and in measuring blood flow in the target splanchnic area, blood flow of pelvic and other organs cannot be excluded. Respiration and posture related to alterations in BF_Ab _should be taken into account when measuring the three arteries. Determination of BF_Ab _by evaluating three-conduit arterial hemodynamics using the technique described in this review may provide a valid measurement that encompasses the comprehensive physiologic arterial blood inflow to multiple abdominal organ systems.

## Abbreviations

BF: Blood flow; Ao: Upper abdominal aorta; LFA: Left femoral artery; RFA: Right femoral artery; BF_Ao_: Blood flow in the upper abdominal aorta; BF_RFA_: Blood flow in the right femoral artery; BF_LFA_: Blood flow in the left femoral artery; FAs: Both femoral arteries; BF_Ab_: Comprehensive blood flow in the lower abdomen.

## Competing interests

The author declares that they have no competing interests.
